# 763 Characteristics and clinical outcomes in patients with combined burn and trauma in Japan

**DOI:** 10.1093/jbcr/irac012.316

**Published:** 2022-03-23

**Authors:** Yasuaki Kumakawa, Yutaka Kondo, Kenshi Suwa, Satoshi Takizawa, Yohei Hirano, Koichiro Sueyoshi, Ken Okamoto, Hiroshi Tanaka

**Affiliations:** Department of Emergency and Critical Care Medicine, Juntendo University Urayasu Hospital, Urayasu,, Chiba; Department of Emergency and Critical Care Medicine, Juntendo University Urayasu Hospital, Urayasu, Chiba; Department of Emergency and Critical Care Medicine, Juntendo University Urayasu Hospital, Urayasu, Chiba; Department of Emergency and Critical Care Medicine, Juntendo University Urayasu Hospital, Urayasu, Chiba; Department of Emergency and Critical Care Medicine, Juntendo University Urayasu Hospital, Urayasu, Chiba; Department of Emergency and Critical Care Medicine, Juntendo University Urayasu Hospital, Urayasu, Chiba; Department of Emergency and Critical Care Medicine, Juntendo University Urayasu Hospital, Urayasu, Chiba; Department of Emergency and Critical Care Medicine, Juntendo University Urayasu Hospital, Urayasu, Chiba

## Abstract

**Introduction:**

Patients with combined burn and trauma are common in the United States and combined trauma in the burn increase the mortality. On contrast, their characteristics and outcomes remain unknown in Japan. The aim of present study was to elucidate the characteristics of trauma combined burn in Japan.

**Methods:**

A multicenter retrospective study was performed between 2004 and 2017 using data from the Japan Trauma Data Bank (JTDB). We evaluated characteristics in burn patients (n=5,783) by segregating them into two groups cohorts: burn only (n=5,537) and combined burn and trauma(n=246). Clinical characteristics such as patient background, severity of burn and trauma, mechanism of injuries, total body surface area (TBSA), injury lesion, treatment and outcome were examined.

**Results:**

The results showed significant differences in age between the burn only group and combined burn and trauma group (median [IQR]: 61 [43–76] for burn only group vs 51 [39–66] for combined burn and trauma group, p < 0.001). Most patients were injured due to flame (burn only group, 65.7%; combined burn and trauma group, 54.9%). Furthermore, 40-89% TBSA was higher in burn only group (19.3%) than in the combined burn and trauma group (9.3%). In-hospital mortality in burn only group (18.2%) was higher than that in the combined burn and trauma group (6.9%).

**Conclusions:**

We demonstrated that the characteristics of burn only and combined burn and trauma patients in Japan. Flame was main cause of burn and in-hospital mortality was higher in burn only group because of higher burn area.

